# Assessment of Tumor Relative Biological Effectiveness in Low-LET Proton Irradiation

**DOI:** 10.3390/biomedicines13081823

**Published:** 2025-07-25

**Authors:** Ying-Chun Lin, Jiamin Mo, Yuan-Hao Lee

**Affiliations:** 1 Department of Radiation Oncology, China Medical University Hospital, Taichung 40402, Taiwan; mark_meg@livemail.tw; 2 School of Medicine, College of Medicine, China Medical University, Taichung 40402, Taiwan; 3 Department of Biomedical Imaging and Radiological Science, China Medical University, Taichung 40402, Taiwan; 4 Department of Radiation Oncology, Chang Bing Show Chwan Memorial Hospital, Changhua 50544, Taiwan

**Keywords:** linear energy transfer, relative biological effectiveness, spread-out Bragg peak

## Abstract

**Background/Objectives**: Within the range of spread-out Bragg peak (SOBP), LET (linear energy transfer) gradually increases from proton beam entrance point toward the beam exit direction. While it is expected that the change in LET would lead to correspondent change in RBE (relative biological effectiveness) on many human cell lines, the incomplete cell killing due to low LET can result in tumor recurrence. Hence, this study aimed to assess the RBE on different cancer cell lines along low-LET proton SOBP. **Methods**: The clonogenicity of A549 and Panc-1 cells after irradiation was evaluated for investigating cell radiosensitivity in response to different types of radiation. The isoeffect doses of 6-MV photon and low-LET proton beams that resulted in equivalent cell surviving fractions at proton dose of 2 or 4 Gy were compared. **Results**: Ratios of α/β of A549 and Panc-1 cells from photon irradiation are 51.69 and −0.7747, respectively; RBE (2 Gy proton SOBP) on A549 and Panc-1 cells are 0.7403 ± 0.3324 and 1.0986 ± 0.3984, respectively. In addition, the change in RBE with proton LET was in a cell-specific and dose-dependent manner (LET-RBE linear correlations: A549 cells [*r* = 0.4673, *p* = 0.2430] vs. Panc-1 cells at 4 Gy [*r* = 0.7085, *p* = 0.0492]; Panc-1 cells at 2 Gy [*r* = −0.4123, *p* = 0.3100] vs. 4 Gy [*r* = 0.7085, *p* = 0.0492]). **Conclusions**: Compared with A549 cells, Panc-1 cells present greater resistance to low-LET proton beams. In addition, currently employed generic RBE value at 1.1 for proton therapy neglected the variation in cell-/tumor-specific radiobiological responses toward different dose levels of proton beams.

## 1. Introduction

The interaction between ionizing radiation and tissues can lead to various biological effects, including hormetic effects at lower doses (<100 mGy) and genotoxic effects at higher doses [1,2,3,4]. As radiotherapy aims at killing tumors while sparing organs at risk (OARs), the efficacy of maximizing the tolerance dose has been undertaken with advances in radiotherapy treatment planning and delivery, such as dose escalation using altered fractionation and stereotactic body radiotherapy (SBRT) [5,6]. On the other hand, radiation-induced severe complications in 5% and 50% of patients within five years post-radiotherapy have been investigated for minimizing radiotoxicity in compliance with the principle of ALARA (as low as reasonably achievable) and for setting up the tolerance doses of OARs (organs at risk), respectively [6,7,8,9]. Nevertheless, the tumor’s response to different types of radiation and/or radiotherapy regimens needs to be better understood in order to carry out radiotherapy treatment planning as effectively as reasonably achievable [10,11]. In reference to studies showing that different tumor control rates can be resulting from differed radiosensitivity of each cell type as well as that the same type of tumor cells responds differently to different types of radiation in cell death signaling [12,13], improved understanding of radiation-induced bioeffects on tumor cells of different origins or different characteristics becomes fundamentally important for developing strategies to combine radiotherapy with other treatments.

In proton therapy, the responses of tumors to radiation are not only related to the total dose but also to the LET (linear energy transfer) of the beams at the same dose rate [14,15]. Given that the distribution of LET is strongly associated with target tissue composition and the organ structures, adequate designs of proton irradiation jigs for achieving clinically relevant values of LET can facilitate the reflection of comparable responses of different types of tumors to radiation used in radiotherapy [2]. In this study, a multi-step irradiation jig was designed specifically for multiplexed irradiation of one pancreas ductal adenocarcinoma (p53 mutated) and one non-small cell lung cancer (p53 wild-type) cell lines in 96-well plates. By investigating the differences in relative biological effectiveness (RBE) calculated from isoeffect doses of photons and the protons over the region of SOBP, we tested whether a currently employed RBE value of 1.1 for proton therapy for all kinds of tumors is related to negligence in the intrinsic disparity among normal and tumor tissues/cells as well as the existence of tumor cell-specific radiobiological responses toward different types of radiation [16,17].

## 2. Materials and Methods

### 2.1. Cell Culture

Authenticated and contamination-free A549 (# CCL-185) and Panc-1 (# CRL-1469) cells were cultured in 96-well plastic plates (Cat. No. 25-109, GenClone, Genesee Scientific, San Diego, CA, USA) using DMEM (Dulbecco’s modified Eagle’s medium, Cat. No. D5796, Sigma-Aldrich, Burlington, MA, USA) supplemented with 10% FBS (fetal bovine serum), 10 units/mL Penicillin, and 1 µg/mL streptomycin at 37 °C in a humidified atmosphere containing 5% CO_2_ in the air. The medium was changed every two to four days and the cultures were split using 0.25% trypsin. All experiments were conducted with cell passage numbers less than 20.

### 2.2. Photon Beam Irradiation

Cell irradiation was performed under normoxia with a TrueBeam linear accelerator (Varian Medical Systems, Inc., Palo Alto, CA, USA). With photon output energy at 6 MV and the dose buildup with plastic water and backscatter materials, cells were irradiated for 1, 2, 3, 4, 5, or 6 Gy.

### 2.3. Proton Beam Irradiation

**Figure 1:**
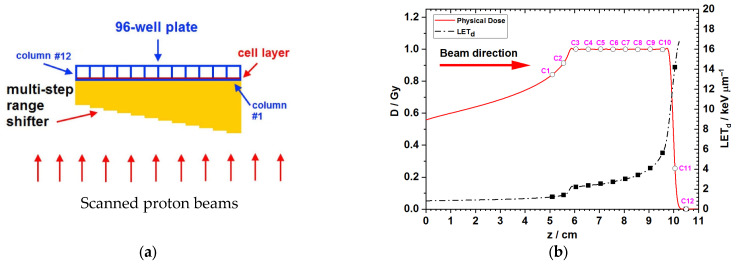
Setup of cell irradiation and Monte Carlo simulations for the dose and LET spectra. (**a**) Schematic illustration of the 12 columns of the 96-well plate in correspondence to the location of the 12 steps of the irradiation jig. (**b**) Dose and LET distributions in the cell layers computed using Monte Carlo. The proton SOBP was developed in area corresponding to the well column numbers 3 to 10 (C3–C10) of the 96-well plate. Note: D, dose; LET_d_, dose-averaged LET; z, distance along the beam direction; c1–12, the first (1 left) to the last (the most right) column of a 96-well plate.

Cell irradiation along the proton SOBP was performed under normoxia with the scanning beam gantry of the synchrotron and the ProBeat proton scanning beam delivery system (Hitachi, Ltd., Tokyo, Japan). The Monte Carlo simulations, physical setup, and irradiation method have been described previously [18]. In brief, Monte Carlo simulations were performed based on the Geant4 toolkit using passively scattered proton beams at accelerated energy ranges from 88.0 MeV to 118.6 MeV. The adopted proton beams generated a 4 cm SOBP as indicated in Figure 1b. A multi-step irradiation jig (also known as. a multi-step range shifter) made of Lucite (ρ = 1.19 g/cm^3^) for the column-by-column simultaneous irradiation of cells in the 96-well plate was used for modulating the LET (2.2–5.6 keV/μm) distribution of proton beams. Cells in the single field flat SOBP were irradiated at doses of 2 or 4 Gy.

### 2.4. Clonogenic Assay

Clonogenicity of cell lines was determined using plate colony formation assay. Cells were seeded onto 96-well plates (Cat. No. 25–109, GenClone, Genesee Scientific) in the quantity of 100 cells per well for irradiation. After irradiation, cells were incubated for up to 14 days and formed into colonies. Colonies then were washed with PBS, fixed with 4% paraformaldehyde for five minutes and stained with crystal violet for one minute at room temperature. Images were captured with ChemiDoc MP Imaging System (Bio-Rad, Hercules, CA, USA). The colony number in each well was assessed using ImageJ (ImageJ 1.53e).

### 2.5. Image Analysis and Survival Curve Fitting

By generating a binary image through thresholding colony images with the Yen method, lightly stained colonies with crystal violet were filtered out prior to further processing with the particle analyzer. Heavily stained colonies in the size bigger than four pixels (covering 50 or more cells) were counted as one colony. Curves of cell survival in response to photon irradiation at different doses were fitted with the linear quadratic equation in GraphPad Prism (Version 8.0.0, GraphPad Software). The nonlinear fit values α, β, and α/β were also obtained from the curve fitting.

### 2.6. Statistical Analyses

All experiments were performed at least in eight replicates. Data of clonogenic survival were analyzed with GraphPad Prism; two-sample *t*-test (two-tailed) was applied for comparisons. The linear relationship between LET and RBE for each cell line was assessed by Pearson correlation (two-tailed). All statistics were expressed as mean ± standard deviation in the text. The *p*-value for all statistical tests was set at <0.050.

## 3. Results

### 3.1. Monte Carlo Simulations for the Dose and LET Spectra Along the Beam Direction

By simulating the attenuation of proton energy in a stepwise fashion with a 12-step jig, the amount of radiation deposited to the cell layer of the 96-well plate, as well as the incurred LET, was grouped into 12 columns, of which SOBP occurred from the column #3 to the column #5, while the LET was increasing from the column #1 to the column #11 (Figure 1, Table 1).

### 3.2. Varied Cell Responses to Photon Irradiation

**Figure 2:**
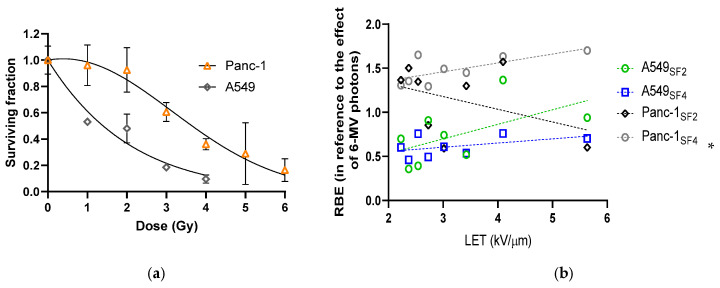
Clonogenics and the assessed surviving fractions and RBE of cells after irradiation. (**a**) Survival curves of A549 and Panc-1 cells from photon irradiation; (**b**) linear relationships assessed between proton LET and RBE for different cell lines at different doses. Note: * indicates statistical significance (*p* < 0.050); A549_SF2_, the values of A549 cells based on the surviving fractions at 2 Gy proton irradiation; A549_SF4_, the values of A549 cells based on the surviving fractions at 4 Gy proton irradiation; Panc-1_SF2_, the values of Panc-1 cells based on the surviving fractions at 2 Gy proton irradiation; Panc-1_SF4_, the values of Panc-1 cells based on the surviving fractions at 4 Gy proton irradiation.

From the shapes of fitted survival curves, the radiosensitivity of A549 cells at the SOBP were significantly higher than that of Panc-1 cells (surviving fractions of A549 vs. Panc-1 cells at 2 Gy: 0.5013 ± 0.1127 vs. 0.8136 ± 0.1019, *p* = 0.012), leading to a relatively larger α/β (Figure 2a, Table 2).

### 3.3. Cell Survival and the Calculated RBE Along and Around the Proton SOBP

Proton-afflicted cell survival was assessed at 2 and 4 Gy with no statistical significance between investigated cell lines (Supplementary Figures S1 and S2, Table 1). Nevertheless, the calculated RBE along the proton SOBP of Panc-1 was significantly correlated with the LET at 4 Gy (Figure 2b, LET-RBE correlation on A549 cells at 2 Gy [*r* = 0.5651, *p* = 0.1444] vs. 4 Gy [*r* = 0.4673, *p* = 0. 4302]; LET-RBE correlation on Panc-1 cells at 2 Gy [*r* = −0.4123, *p* = 0.3100] vs. 4 Gy [*r* = 0.7085, *p* = 0.0492]).

## 4. Discussion

In the early study of Gerweck and Kozin, the RBE of SOBP protons was found to increase with decreasing dose and are likely to be dependent on the α/β of the target cells or tissues [19]. This inverse relationship between the RBE of SOBP protons and radiation dose is also manifested from the clonogenics of cell lines that were applied in this study. The differed cell/tissue specificity in radiosensitivity was also found from clinical radiotherapy studies [20]. Regarding the wide range of the α/β among different tumors, the corresponding RBE is hence anticipated to be varied across different tumors. From the experimented cell lines of this study, the RBE of A549 over the SOBP was calculated to be lower than 1.0 (the overall RBE reduction rates for 2 and 4 Gy proton irradiation were 26.0% and 38.4%, respectively). This result reveals the uniqueness of genetic control of radiosensitivity toward different types of radiation for each cell line. It is known that tumors harboring mutated genes that are involved in DNA damage response and cell death signaling may aggravate or prevent cells from necrotic, mitotic, and/or apoptotic death as well as senescence after irradiation [21]. From the fitted α and β parameters of the linear-quadratic models, x-ray-induced DNA single-stranded breaks were shown to result in greater cell lethality on A549 cells. On the other hand, it is suspected that the sublethal DNA damage induced by proton beams at clinically relevant low levels of LET along the SOBP was repairable in p53 wild-type A549 cells as compared with p53-mutated Panc-1 cells, leading to the smaller RBE in reference to the killing effect of 6-MV energetic photons [13,21,22].

In contrast to the other observation obtained from normal cell and tissue studies by Gerweck and Kozin [19], we found that the RBE along the proton SOBP slightly increased with increasing dose for the pancreatic tumor cell line. The contradictory result reveals that the susceptibility of pancreatic tumor cells to low-LET proton beams become much more marked than that to 6-MV photon beams at higher doses. This result might be attributed to the functional base excision repair machinery of Panc-1 cells for repairing single-stranded breaks but deficiency in p53-mediated DNA damage response to double-stranded breaks [23,24]. Nevertheless, this dose-RBE relationship was reversed on the non-small cell lung cancer cell line, indicating that SOBP proton-induced dose response of A549 cells (resembling the early responding tissues) is more phenotypically like that of the normal cells/tissues than that of Panc-1 cells (resembling the late responding tissues). In addition, moderately positive correlations were found between LET and RBE on A549 cells at different doses whereas the correlations on Panc-1 cells were moderately negative at 2 Gy and strong positive at 4 Gy, respectively. These results suggest that the RBE for tumor cells along the SOBP can be changed not only by cell characteristics but also by the LET and dose of the proton beams.

Even though high levels of standard deviations were recorded when assessing the RBE of different tumor cells along the SOBP as well as the surviving fractions of tumor cells after photon irradiation, significant correlation between proton LET and RBE on Panc-1 cells at 4 Gy, as well as significant difference in SF between A549 and Panc-1 cells, was still discovered. This study shows that the interplay of dose and LET can significantly affect biological outcomes, suggesting a need for more personalized treatment planning (including parameters like fraction size and LET distribution). As a result, the clinically adopted RBE, 1.1, cannot be utilized for correctly predicting cell lethality in response to low-LET proton irradiation.

## 5. Conclusions

In conclusion, the generic RBE at 1.1 may not be justifiable on tumors of genetic inhomogeneity and instability when applying low-LET proton beams. Further assessments of the overestimated RBE of low-LET protons on 3D co-cultured cell models (composed of non-cancerous and cancerous cells to mimic the physiological and pathological conditions of solid tumors under irradiation) will facilitate maximizing the therapeutic benefit of proton therapy through an enhanced database for treatment planning.

## Figures and Tables

**Table 1:** Cell survival variations and average RBE over the range of the SOBP. Abbreviations: SF, surviving fractions; SF2, surviving fractions at 2 Gy; SF4, surviving fractions at 4 Gy. Note: * indicates statistical significance (*p* < 0.050) when compared with SF2.

Column #	Relative Dose Modulation Factor	LET (keV/μm)	Surviving Fractions of A549 Cells		Surviving Fractions of Panc-1 Cells	
			SF2	SF4	SF2	SF4
1	0.8415	1.2269	0.7272 ± 0.3517	0.6818 ± 0.4573	0.7917 ± 0.2077	0.4167 ± 0.1460
2	0.9131	1.4134	0.9787 ± 0.4724	0.6064 ± 0.3943	0.7907 ± 0.2886	0.5000 ± 0.1990
3	1.0009	2.2267	0.5000 ± 0.2757	0.2963 ± 0.2726	0.7027 ± 0.2694	0.2162 ± 0.2754
4	1.0004	2.3710	0.7045 ± 0.3489	0.3977 ± 0.4232	0.6429 ± 0.2919	0.1905 ± 0.2215
5	1.0006	2.5418	0.6809 ± 0.3975	0.2128 ± 0.1712	0.7105 ± 0.2190	0.0789 ± 0.1878
6	0.9997	2.7281	0.4043 ± 0.2461	0.3723 ± 0.5227	1.1111 ± 0.3440	0.2222 ± 0.2257
7	1.0002	3.0143	0.4792 ± 0.2645	0.2917 ± 0.2893	1.0323 ± 0.4057	0.1290 ± 0.2489
8	0.9992	3.4235	0.6000 ± 0.4695	0.3400 ± 0.3974	0.7317 ± 0.1987	0.1463 ± 0.1807
9	1.0007	4.0951	0.2500 ± 0.1792	0.2115 ± 0.1956	0.6111 ± 0.2150	0.0833 ± 0.1177
10	0.9978	5.6344	0.3913 ± 0.2731	0.2391 ± 0.2507	0.9667 ± 0.3012	0.0667 ± 0.1331
11	0.2549	14.1959	0.5227 ± 0.3763	0.3750 ± 0.3141	0.7429 ± 0.3620	0.5143 ± 0.2590
12	7.94 × 10^−4^	4.15 × 10^−6^	0.7843 ± 0.3894	0.9804 ± 0.4558	1.1860 ± 0.5081	0.8605 ± 0.2584
Average SF over the range of SOBP	0.9999 ± 0.0010	3.2544 ± 1.1389	0.5013 ± 0.1127	0.2952 ± 0.1185 *	0.8136 ± 0.1019	0.1416 ± 0.0726 *
RBE in the range of SOBP	------	------	0.7403 ± 0.3324	0.6158 ± 0.1158	1.0986 ± 0.3984	1.4608 ± 0.1620

**Table 2:** Fitted parameters from analyses of the linear-quadratic models in Figure 2a.

	Cell Lines	A549	Panc-1
Parameter			
α (95% CI)		0.4822 (0.2930, 0.6831)	−0.05115 (−0.1621, 0.0579)
β (95% CI)		0.0093 (−0.0602, 0.0886)	0.0660 (0.0363, 0.1013)
α/β		51.69	−0.7747
R squared		0.9235	0.8629
Degrees of Freedom		18	26

## Data Availability

The original contributions presented in this study are included in the article. Further inquiries can be directed to the corresponding authors.

## Supplementary Materials

The following supporting information can be downloaded at https://www.mdpi.com/article/10.3390/biomedicines13081823/s1. Figure S1: Representative wells of colonies formed by A549 cells in the 96-well plate at different proton irradiation doses. Figure S2: Representative wells of colonies formed by Panc-1 cells in the 96-well plate at different proton irradiation doses

## Abbreviations

The following abbreviations are used in this manuscript:

LET	Linear energy transfer
OAR	Organs at risk
RBE	Relative biological effectiveness
SOBP	Spread-out Bragg peak
