# Supplementary material for: Assessment of Tumor Relative Biological Effectiveness in Low-LET Proton Irradiation
Source: Biomedicines. 2025 Jul 25;13(8):1823. doi: 10.3390/biomedicines13081823 (PMC12383938; doi:10.3390/biomedicines13081823)
Supplement: Supplementary file 1 [file biomedicines-13-01823-s001.zip › biomedicines-3713549-supplementary.pdf]

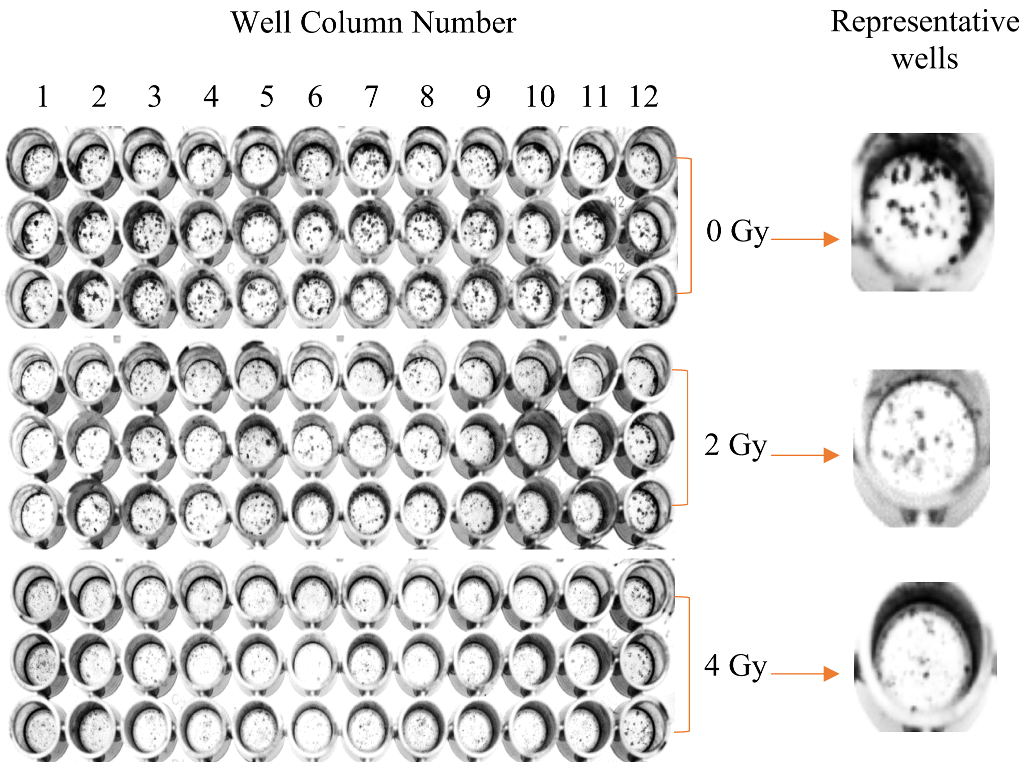

Supplementary Figure S1. Representative wells of colonies formed by A549 cells in the 96-well plate at different proton irradiation doses.

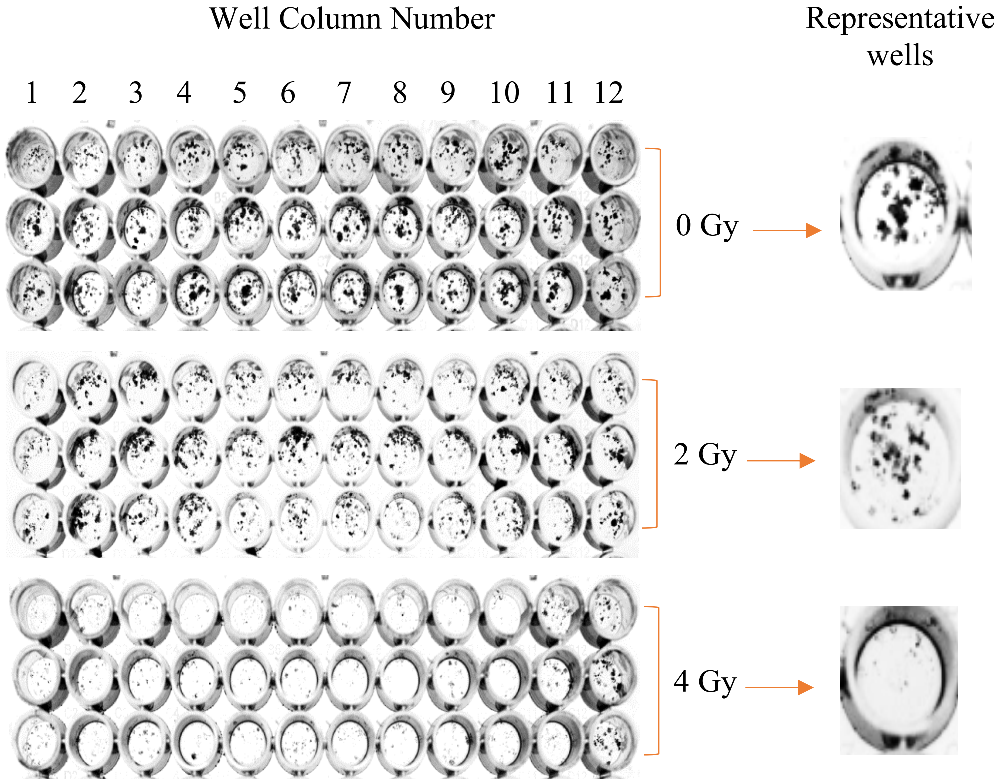

Supplementary Figure S2. Representative wells of colonies formed by Panc-1 cells in the 96-well plate at different proton irradiation doses.
